# Clinical artificial intelligence applications of vision-language foundation models

**DOI:** 10.1371/journal.pdig.0001453

**Published:** 2026-06-11

**Authors:** Arun James Thirunavukarasu, Siyou Li, Pengyao Qin, Dong Nie, Rohan Sanghera, Ernest Lim, Juntao Yu, Le Zhang

**Affiliations:** 1 Department of Clinical Neurosciences, Medical Sciences Division, University of Oxford, Oxford, United Kingdom; 2 International Centre for Eye Health, London School of Hygiene and Tropical Medicine, London, United Kingdom; 3 School of Electronic Engineering and Computer Science, Queen Mary University of London, London, United Kingdom; 4 School of Engineering, College of Engineering and Physical Sciences, University of Birmingham, Birmingham, United Kingdom; 5 Meta AI, Meta Platforms Inc., Menlo Park, California, United States of America; 6 Heidi Health, Melbourne, Australia‌‌‌‌‌‌‌‌; 7 Institute for Safe Autonomy, University of York, York, United Kingdom; 8 Ufonia Ltd., Oxford, United Kingdom; 9 Moorfields Eye Hospital NHS Foundation Trust, London, United Kingdom; Ben-Gurion University of the Negev, ISRAEL

## Abstract

Vision-language models (VLMs) represent a transformative advance in generative artificial intelligence (AI), using multimodal data processing to enhance clinical decision-making and workflow efficiency. Built on transformer architectures, VLMs excel in tasks like image interpretation, report generation, and visual question-answering, with emerging applications in radiology, pathology, and broader clinical practice. Their potential extends to automating documentation, improving medical education, and assisting with clinical decision-making in real-time. However, successful integration requires rigorous validation to address challenges such as bias, interpretability, and safety concerns. Prospective clinical trials, health economic evaluations, and stakeholder engagement are essential to ensure equitable and effective deployment. Regulatory frameworks must evolve to accommodate VLM functionality while maintaining accountability and protecting patient safety. By balancing innovation with robust oversight, VLMs hold promise in reducing clinician workload, expanding access to expert care, and advancing precision medicine—ushering in a new era of AI-augmented healthcare.

## Introduction‌‌

The advent of generative artificial intelligence (AI) with remarkable abilities to respond appropriately and flexibly to diverse queries from human users has generated interest in potential clinical applications [1]. Interest was initially piqued by large language models (LLMs), as exhibited by uptake of chatbot applications such as ChatGPT, Google Gemini, and Claude [2]. Further development has generated models that can process multimodal data, including images, expanding the functionality and potential applications of these systems [3]. AI models that can process and produce images and text are known as vision-language models (VLMs) and include the latest generations of multimodal chatbot applications.

Many terms are used to describe generative AI models, which can cause ambiguity and confusion. ‘Foundation model’ is a broad umbrella term capturing AI that is pre-trained on large volumes of unlabelled data before fine-tuning on labelled data (*e.g.,* conversational input and output text, or annotated images). Foundation models exclusively trained on text data are known as LLMs, and represent the earliest widely successful application of this pre-training and fine-tuning schema [2]. As these schemata have begun to incorporate multimodal data (*e.g.,* text, images, tables, sound, and video), reference has been made to ‘multimodal LLMs’, but this is an oxymoron [4]. For foundation models that process language and images, VLM is a preferable term [5].

Clinical practice is highly multimodal, involving information gleaned from spoken conversation and physical examination, captured through laboratory and imaging investigations, and documented in free text and tabular formats. Through their inherent multimodality, VLMs offer opportunities to reduce the workload borne by clinicians and even to expand the capabilities and function of healthcare professions [3]. Extensive work evaluating the potential of text-based LLMs has already been undertaken, and multimodal applications are likely to offer broader functionality and utility in healthcare settings [6,7].

In this narrative review, the technical underpinnings of VLMs are discussed and their potential applications in healthcare are explored. The validation pathways that VLM researchers might use to gain acceptance in clinical practice are outlined, with an emphasis on robust clinical research to justify clinical interventions. Barriers to development and implementation are considered, with specific assessment of diverse stakeholders’ perspectives. As VLM applications remain nascent in routine clinical practice, examples from adjacent technologies are drawn upon in the discussion. VLM developers and clinicians working together are well placed to engineer interventions that can improve the provision of healthcare worldwide; this review provides an informative overview that can help guide the work of interested researchers.

## Technical overview of vision-language models

VLMs leverage ‘transformer architectures’ to integrate textual and audiovisual data to enable tasks like conversational interaction, image interpretation or reporting, visual question-answering, and text-to-image generation [8]. While longer standing architectures can be ensembled in applications that interpret and produce multimodal information—such as image classifiers, generative adversarial networks, and determinative chatbots—these rules-based models do not perform ‘vision-language modelling’. Vision-language modelling entails combining text and image data into a unified representation, which permits automation of more advanced tasks than with applications comprised of discrete modules that process different modalities.

### Transformer architectures and training

**Fig 1:**
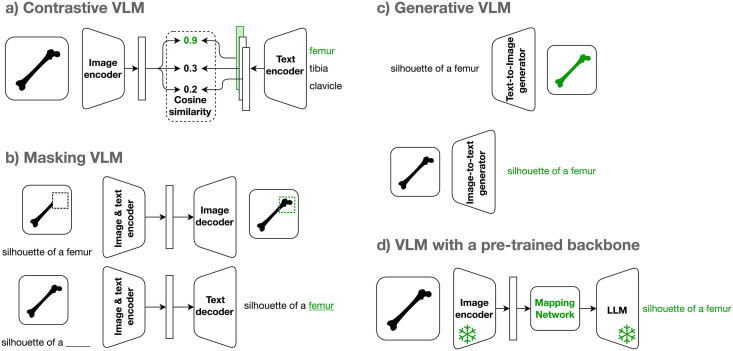
Four architectural paradigms of vision-language modelling. These architectures are not mutually exclusive, and aspects of each are frequently combined with one another. **(A)** Contrastive vision-language models (VLMs) convert image and text inputs into embeddings that are represented in a multimodal representation space, allowing for combination and comparison to fulfil user-defined tasks. **(B)** Masking VLMs are trained to reconstruct images or text with missing portions when provided with multimodal data as a reference. **(C)** Generative VLMs are trained to generate output whole, such as through sequential production of tokens (words) and pixels, or through iterative denoising that can be directed by textual or image prompts. **(D)** VLMs with pre-trained backbones take advantage of existing models—such as LLMs trained and fine-tuned at scales beyond most research teams—to take advantage of innate knowledge and reasoning abilities, which can be combined with multimodal input data.

VLM training aims to confer image and text processing abilities with an appropriate association between data formats. A wide variety of training tasks are used—corresponding to the architectural paradigms in Fig 1—which challenge the VLM to appropriately process matched multimodal information, such as words from passages of free text and pixels from images. As VLM performance improves on these tasks, they develop an ability to produce descriptive text in response to images, or *vice versa* [9,10]. With sufficient training, these capabilities generalise beyond the images within the pre-training dataset, leading to useful capabilities without specific training (‘zero-shot performance’) [11]. Emergence of zero-shot performance is ultimately dependent on scale, with up to hundreds of millions of images and enormous computational resource requirements [9]. Interrogation of these foundation models indicates that their abilities stem from genuine abilities to associate, reason, and plan—rather than mere pattern recognition or matching to previously encountered material [12].

Transformer-based architectures may be grouped into four broad categories, although these architectural and training paradigms are not mutually exclusive and many models exhibit combination and overlap of these methods [8,13]. The first category is contrastive vision-language modelling (Fig 1a). Contrastive VLMs usually consist of a text encoder and an image encoder. These encoders convert inputs (such as free text or images) into ‘embeddings’: representations of information within a unified (multimodal) data-space [14,15]. This facilitates combined processing of text and visual data, which permits direct comparisons of similarity between words and images. Examples within medicine include CXR-CLIP and CT-CLIP, which produce textual reports in response to chest X-ray (CXR) or chest computed tomography (CT) images [16,17]. These models are extensions of CLIP, a popular pre-trained neural network that is trained to map related image-text pairs closer together to facilitate downstream recognition of novel but related objects or scenes without further training [9].

The second category is VLMs trained with masking-based tasks. In this approach, models are challenged to reconstruct masked (hidden or obscured) portions of images when provided with textual captions, or *vice versa*: reconstructing obscured captions when provided an unmasked image (Fig 1b); similar to the pre-training of many LLMs [2]. Masked autoencoders typify this approach: already well established in developing applications that can reconstruct images without textual guidance, and used in a variety of visual foundation models in medicine such as RETFound [18,19]. Once trained, masked autoencoders can be fine-tuned on a wide variety of tasks that do not involve reconstructing images, such as classifying pathology on provided images [19]. Broadening training and fine-tuning to include text-based and other data formats broadens the capabilities of these model, such as by enabling generation of appropriate text in response to images [20]. FLAVA is a frontier VLM that was developed using masking strategies that required less than a fifth of the data used to train CLIP, with a curated but open source set of images and text [21].

Generative vision-language modelling is a relatively recent third category (Fig 1c). While earlier schemata depend on abstract representations produced by encoder components that map images, text, and other data onto a multimodal latent data-space, generative VLMs generate text and/or images more directly. These generative VLMs are trained to produce complete images or phrases in the form of sequences of tokens representing pixels or word fragments—frequently requiring higher computational costs for development [22]. CM3Leon is an instructive example that facilitates image-to-text and text-to-image generation, both from scratch as well as controllable editing at user-defined levels of abstraction [23,24]. Diffusion models are a widespread subset of generative VLMs, which are trained to reverse the process of decomposition into random noise; thereby producing realistic text, images, and other data from scratch [25]. Generative VLM applications include Stable Diffusion, which combines a U-Net diffusion model (for denoising) with a variational autoencoder (for image embedding) and CLIP text encoder (for text-embedding) to facilitate image synthesis in response to textual prompts [22,26,27].

Finally, VLMs can be built using a pre-trained backbone (Fig 1d). As with contrastive VLMs, an image encoder is used to enable representation of images in a multimodal space that can also accommodate textual information. Interfacing with an LLM is used to promote association between images and relevant text [28,29]. The LLM can be fine-tuned for a variety of use cases including report generation, visual question-answering, or segmenting requested organs or lesions—as exhibited by M3D-LaMed which works with three-dimensional imaging modalities such as CT [30]. Other examples include Frozen and MiniGPT, which map representations of images to pre-trained LLMs to guide text production [31,32]. These LLMs offer unparalleled text-embedding capability and contain useful knowledge and reasoning abilities that may transfer to clinical tasks [33–35]. Most research teams do not have the necessary resources to develop comparable models from scratch, making incorporation of a pre-trained LLM attractive. While this entails reliance on an external model with vulnerability to unannounced changes or discontinuation, a growing number of open source models mitigate these risks and exhibit competitive performance [28,29,36].

### Improving alignment through fine-tuning

Once a VLM is trained to generate appropriate-seeming text in response to images, or *vice versa*, further fine-tuning may be undertaken to improve the usefulness of generated content. The aim of fine-tuning is to promote VLM outputs that align with users’ requirements or towards a particular distribution of desired behaviours, and fine-tuning processes can be modified based on the aims of development. Many VLM applications are instruction-tuned: exposed to instructions and other inputs (*e.g.,* free text queries and images) and desired outputs (*e.g.,* image interpretation) [37]. While input/output pairs may be produced by humans, the general requirement for large datasets for fine-tuning has stimulated research into synthetic (generative AI-produced) data, with results exceeding the performance of previous VLMs [38].

Further progress has followed from pursuing similar approaches to those exemplified by chatbot applications [2]. By training an extraneous ‘reward model’ using human ratings of output text or images, a VLM can be autonomously fine-tuned to optimise predicted human ratings of its outputs in response to a large number of input challenges [39]. This ‘reinforcement learning from human feedback’ has been further augmented by using frontier LLMs or VLMs to generate ratings rather than humans, facilitating ‘reinforcement learning from AI feedback’ at an even greater pace [40]. Relying on a reward model that scores an abstract concept of quality is a convenient means of developing VLMs with useful responsiveness and reasoning capabilities, without defining an exhaustive list of metrics or qualities that distinguish good outputs from bad [41].

## Existing and potential applications of vision-language models in medicine

Most commonly, VLMs are applied to imaging data combined with textual or tabular EPR data, although other combinations can include free text from clinicians or patients, omics data (*e.g.,* genetic sequencing), and time-series data including significant clinical events [42]. VLM applications can assist clinicians that specialise in imaging interpretation—such as radiologists and pathologists—or in a wider array of specialities where clinicians conduct, interpret, and act upon imaging as a smaller component of their responsibilities. Considering realistic and useful applications of VLMs—rather than what is currently possible with available data—is essential to direct innovation that leads to positive and impactful change [43]. However, as VLM applications are not yet widely used in healthcare, examples from adjacent technologies are used to illustrate the discussion.

### Radiological and pathological applications

A wide variety of VLM applications are being developed and validated in radiology and pathology, where much of the clinical workload relates to asynchronous image interpretation [44,45]. These applications fit into a variety of models of ‘computer-assisted detection’ (CAD) [46]. The most obvious use cases for VLMs in radiology and pathology relate to replicating the tasks undertaken by specialists, either autonomously or by assisting supervising clinicians [46]. These functions could act to increase productivity and broaden accessibility, thereby reducing wait times and increasing the proportion of patients that receive expert care.

Successful incorporation of non-doctor imaging interpretation—such as radiographers reporting basic X-ray investigations—illustrates where generative AI could feasibly fit within care pathways in acceptable roles to clinicians and patients [47,48]. The latest forms of VLM—using transformer architectures—exhibit unprecedented performance, which could be of greater use than classical deep learning classification systems in clinical environments [49]. In retrospective studies, non-expert doctors using generative AI exhibit comparable performance to expert radiologists in assessing chest radiographs, and are also faster than unaided clinicians [50]. Moreover, VLMs tasked with reporting chest radiographs can be indistinguishable from on-site radiologists and superior to teleradiology physicians when rated by board-certified physicians [51]. Models are emerging which broaden capabilities to more data-intensive cross-sectional imaging such as CT and MRI scans: examples include CT-CLIP and M3D-LaMed, which preserve three-dimensional imaging and generate appropriate textual reports that detail and localise abnormalities [17,30]. However, it is worth noting that these impressive results are generally reported from retrospective studies, often small in scale; performance may well degrade in prospective or multicentre trials [52,53].

Image-based pathology tasks lend themselves to similar approaches to automation as well demonstrated in radiology. Foundation models used ‘out-of-the-box’ or with relatively simple modifications exhibit some ability to interpret pathological features, particularly those corresponding to more common conditions [54]. Many domain-specific foundation models have been developed to improve accuracy and expand functionality, using large datasets of pathology images and corresponding descriptions or reports [55–57]. Tasks that VLMs can assist with range from simple classification (categorising images based on the pathology they feature) to more sophisticated report generation, segmentation or highlighting regions of interest, and producing synthetic images and text for educational or research purposes [58]. A growing number of AI pathology applications have gained regulatory approval, although these tend to be narrow in scope—frequently limited to a single disease group or tissue type [45]. Further work is anticipated to lead to VLM applications with more general capabilities that can offer more assistance to pathologists to mitigate the burden of a growing volume of investigations [59].

### Medical and surgical applications

In medicine and surgery, VLM applications relate more to synchronous patient interaction. Administration and documentation provide a potential low-risk domain for AI intervention. VLMs can automate documentation tasks through summative capabilities, whilst incorporating interpretations from imaging modalities present in the patient’s EHR, consequently improving the representation of these investigations in downstream documentation and decision-making [60]. A growing number of AI scribes and other documentation tools are being used in day-to-day clinical work already [61]. While currently generally limited to audio and text processing, these applications will improve their functionality with capacity to process images. Similarly, clinical education offers another lower-risk environment, and VLMs have demonstrated abilities in question-answering, annotating images, and synthetic data generation which can support development and learning of healthcare professionals [62]. Through explainable visual grounding and simulated data, VLMs can have a substantial positive impact on training through interactive scenarios and dynamic feedback [63].

VLMs also have significant potential to contribute more actively to clinical decision-making. Early evaluations of their potential have used aptitude tests that doctors are expected to pass as part of their training, which require an ability to incorporate clinical images, medical knowledge, contextual information, and semantic clinical reasoning [60,64–66]. These applications establish a technical basis for deploying VLMs as clinical co-pilots to assist with real-time decision-making. Beyond direct question-answering, VLMs could be used in parallel with clinicians as safety nets to mitigate medical errors and provide real-time feedback to clinicians to improve their decisions [67]. Some early studies in clinical settings even suggest that AI plans and suggestions can be superior to clinicians working with the same information [68]. However, it is important to note that most early evaluations rely on unvalidated surrogates of actual clinical utility and safety, and further validation in real-world settings is necessary [69].

**Fig 2:**
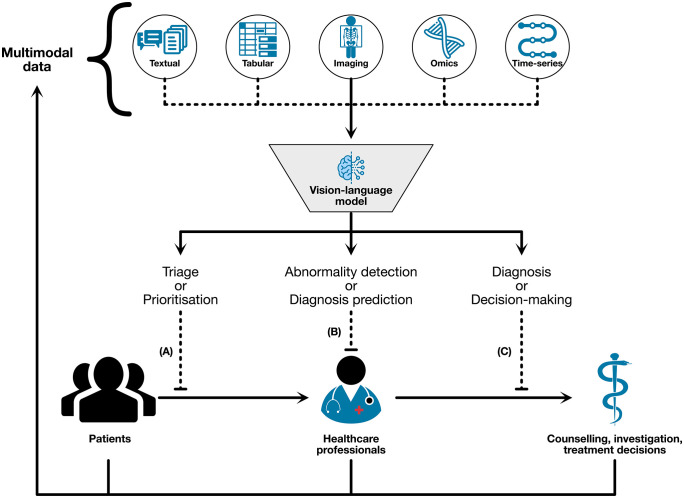
The potential roles of vision-language models in healthcare settings. Artificial intelligence could impact healthcare work through a wide variety of applications, but these may be conceptualised as one of three interactions with clinicians: **(A)** Direction of clinician efforts by prioritising patients based on the predicted severity or urgency of their investigation results; **(B)** Assistance of clinician efforts by augmenting investigation results with predicted diagnoses or risk scores based on recognised pathological features; or **(C)** Replacement of clinician efforts by automating aspects of the clinical workflow, such as by providing diagnoses, patient advice, or making decisions about further investigation, referral, and treatment without direct oversight.

Rather than imitating the work of image-based specialties such as radiology and pathology, VLMs could offer new functionality in specialties where clinicians review images themselves. In resource-constrained environments, it may be unfeasible for subspecialist experts to review every fundus photograph, dermatoscopy picture, or endoscopy recording. However, VLMs trained on a set of labelled images that could be feasibly produced by these experts could provide a resource to offer expert interpretation of images to inform decision-making—much like how physicians and surgeons frequently rely upon reports from radiologists and pathologists (Fig 2B). This could improve efficiency (addressing the growing demand for healthcare) and broaden access to expert-level care [70]. Early examples of applications demonstrating potential in this domain include ophthalmology VLMs for fundus photography and optical coherence tomography (OCT), cardiology VLMs for echocardiograms and electrocardiograms (ECGs), and neurology VLMs for electroencephalogram analysis [71–76].

The proliferation of automated ECG interpretation (generally relying on more basic algorithms than vision-language foundation modelling) serves as a useful analogy; clinician error is concerningly frequent, and while overreliance on imperfect computer-generated reports can lead to mistakes, their ability to highlight nonnormal traces makes them a useful safety net for inexpert clinicians [77,78]. Moreover, the ability of VLMs to output multimodal data—such as bounding boxes or highlighted regions indicating pathological features—could improve the explainability and usefulness of image interpretation for clinicians [79].

Training and fine-tuning with data other than expert labels could empower VLMs to work as risk calculators and treatment response predictors. Unimodal LLMs have already been used to predict clinical events and future diagnoses with remarkable accuracy after training on patient record data, and performance is likely to improve with access to multimodal data including imaging and genomics [80,81]. Predictive VLMs could improve healthcare decisions by identifying patients at higher risk of complications and deterioration, or by suggesting where treatments are likely to have greater or lesser effects. This could facilitate ‘precision medicine’—tailoring treatment to patients’ unique phenotypes, but requires careful study in prospective trials to ensure that patients are not adversely affected by bias or inaccuracy in decisions suggested by VLM output [82].

Finally, VLM applications could interface with patients rather than clinicians to provide clinical advice and reduce the demand for specialist services. A growing number of studies have evaluated the ability of AI chatbots to provide evidence-based medical advice and guidance [6]. Comparisons of an LLM application with physicians in textual real-time conversations with patient actors suggest that AI can provide superior diagnostic accuracy and conversational quality [83]. As many patient triage services rely on inexpert providers applying rigid algorithms—such as call centre professionals in the NHS 111 triage service or receptionists in primary care—there is great scope to expand access to more flexible and expert conversationalists. Dora R1, an autonomous AI chatbot, is successfully used for autonomous telephone follow-up of ophthalmology patients after surgery, and demonstrates how validated applications can reduce clinicians’ workload—perhaps with great potential to impact if able to incorporate image data as well as audio and text [84]. These applications could prioritise access to appointments and direct referrals (Fig 2A), or even initiate investigation and treatment for conditions where the risks related to false positive and false negative classification are acceptable (Fig 2C).

## Validation pathways for vision-language models

### Preclinical validation

Reliance on similar data to that used in training and fine-tuning is insufficient due to risks of overfitting—performance optimised for a single dataset may not generalise in new contexts where idiosyncratic differences in imaging technique and population may generate subtly different distributions of features [85]. External validation—using data to which models have not been previously exposed—is therefore essential [86,87]. A growing number of datasets are publicly available to facilitate validation, but further work is needed and developers may need to gather new data specific to their proposed use case, particularly for complex multimodal interventions or applications where augmentation of data (such as bounding boxes around pathological features) is needed [88,89]. Although patient data is frequently difficult to gather due to privacy concerns, researchers have gathered imaging and text data from novel internet sources such as social media, which has been sufficient for development and robust validation exercises [55,90].

The type of application should inform the design of validation study and evaluation metrics used to quantify performance. Frequently, linguistic metrics borrowed from computer science have been used for their convenience, but these fail to capture clinical accuracy and usefulness [91]. Examination performance has also been used frequently, but this requires contextualisation and has not been shown to predict usefulness in real-world settings [33]. Many studies evaluating AI advice fail to define a reference standard or use subjective judgement without referring to evidence-based guidelines [6]. Ideally, performance evaluation should capture the success of VLM applications concisely and intuitively, and should also specifically explore the frequency and potential consequences of failure and edge-cases [92,93]. To mitigate the problems of *p*-hacking and publication bias, researchers should be encouraged to define their evaluation and analytical plan prospectively and disseminate their findings regardless of their significance (such as through a preprint server). Making models open for others to use can allow external researchers to undertake independent validation studies to verify generalisability of performance.

### Clinical validation

Once an application has shown good potential with a clear conceptualisation of how it may be implemented in healthcare settings, clinical validation is essential. Close oversight may be especially necessary during validation to ensure that the risk of harm to patients is minimised. For interventions determining diagnosis, investigation, and treatment, randomised clinical trials may be required to provide objective evidence of the effectiveness and safety of the system [69]. Previous trials of AI interventions have been small in scale (often limited to single centres), relied on nonclinical or unvalidated surrogate endpoints, and lacked comprehensive demographic data–factors that make it challenging to assess the generalisability of results [52,94]. Conducting larger studies with clinical primary endpoints based on morbidity and mortality, as well as improved transparency in reporting, would provide more robust evidence supporting the deployment of GAI in clinical settings. To ensure that systems do not generate harm, which may not be captured in trials powered to detect differences in primary outcomes that are more frequent than adverse safety events, structured revalidation and ongoing monitoring for adverse effects may be organised, analogous to stage 4 clinical trials [95].

For nonclinical interventions designed to enhance clinicians’ productivity or overall work experience, formal clinical trials may not always be essential [69]. However, prospective randomisation remains the most reliable approach to establishing the causal effects of new interventions, and A/B testing has long been applied outside medicine [96,97]. Reliably assaying the benefit or lack thereof of digital health interventions is essential because previous changes, such as electronic patient records have led to inefficiency, lower-quality documentation, and clinician burnout despite their intended advantages [98,99].

Beyond validation of clinical and operational benefits, health economic analysis is a key step before deployment to ensure that limited resources are directed towards interventions that confer maximal gain to patients and practitioners [100]. This is especially important for systems that require significant resource investment due to opportunity costs; comparisons with alternative interventions can help ensure that changes maximise benefit to patients [101]. Illustrative examples from previous generations of AI include high performance diabetic retinopathy screening, which exhibited optimal cost effectiveness with a ‘human-in-the-loop’ rather than autonomous use in a Singaporean study [100]. Additionally, ethical concerns such as bias and fairness must be addressed, and several initiatives have been established to support clinicians, researchers, and policymakers in actively mitigating these challenges [102–104]. Through standardised, high-quality work in these areas, the field can continue progressing toward greater fairness in AI-driven healthcare solutions.

## Barriers to validation and deployment

### Utilitarian concerns

The primary aim of clinical interventions is to improve healthcare accessibility and effectiveness, thereby optimising patient outcomes and minimising harm. These utilitarian concerns require careful validation and monitored implementation of VLM applications, as plausible changes to healthcare can nevertheless worsen outcomes, either directly due to poor real-world performance or indirectly by representing an opportunity cost that prevents more useful interventions being pursued [105]. Reporting guidelines have been established to promote rigorous and transparent research to maximise generalisability of AI validation studies, and include CHART (for generative AI providing clinical advice), DECIDE-AI (for decision-support systems) as well as TRIPOD-LLM and TRIPOD-AI (for prediction models) [93,106–108]. Supplements to quality assessment tools to improve critical appraisal in systematic review or other evidence synthesis have also been developed, such as PROBAST-AI (for prediction models) [92].

Research has identified a particularly concerning bias in VLMs, where ethnic or gender disparities in diagnostic accuracy lead to the underdiagnosis of underserved patient populations [109–111]. Studies have revealed significant true positive rate (TPR) disparities across various protected attributes, including race, gender, and socioeconomic factors, with real-world implications that can lead to inequitable and unfair outcomes for patients [112]. It is possible that biological differences define differential performance ceilings even with optimised performance of human-delivered or AI-powered healthcare, but further work nevertheless remains necessary to address these disparities.

Beyond bias, the use of VLMs in clinical practice raises several other safety concerns, including the potential for erroneous clinical decisions. Textual output from VLMs, often generated by component LLMs, can generate plausible-sounding but factually incorrect or fabricated information (‘hallucinations’), which can lead to inappropriate medical decisions [2,113]. Additionally, shortcut learning, where the AI system relies on superficial or irrelevant features in the data, can severely impact diagnostic reliability and lead to poor performance in real-world clinical settings [114]. Even with accurate and reliable VLMs, the misinterpretation of model outputs by clinicians who over-rely on predictions without understanding their limitations can result in suboptimal clinical decisions [115].

Addressing these utilitarian concerns and developing strategies to mitigate bias and safety issues are crucial for the fair and responsible integration of VLMs into healthcare.

### Deontological concerns

Deontological concerns stem from risks of breaching moral duties such as transparency, accountability, and respect for human autonomy. The ‘black-box’ nature of frontier VLM architectures complicates verification of AI suggestions [116]. This opacity conflicts with the principle of informed clinical judgment, directed by accountable clinicians and patients’ priorities and values. For instance, a VLM may diagnose a rare condition based on subtle imaging features, but without providing a decision pathway that can be traced to establish plausibility. In addition, accountability for actions taken on the basis of recommendations that may be erroneous has not been established, although the simplest default recourse is for clinicians to retain all responsibility when using tools such as VLM applications [117]. This responsibility should come with autonomy to disregard model suggestions if they are not felt to be useful, as clinicians otherwise risk losing their ability to practice independently and having their performance inextricably linked to AI [118].

Current consent processes rarely address the role of AI in decision-making, and need to be updated [119]. Patients have a right to know if their care is informed by VLMs, and especially if their data is planned to be used for further model development and validation. Ideally, patients should be offered the chance to opt-out of AI-informed care or use of their data for training. Some stakeholders go further, advocating for explicit opt-in schema requiring patients to give informed consent for any use of their data [120,121]. Conversely, others advocate for a civic duty to provide anonymised data to advance development and produce more useful models to improve healthcare for all [122]. Opt-out schemata may represent an acceptable middle ground that preserve patient autonomy while maximising availability of data to promote innovation [123].

### Stakeholders’ concerns

Ultimately, AI applications must be acceptable to users and appropriately regulated with oversight that addresses deontological and utilitarian concerns. Users may be clinicians using applications for decision-support or improved efficiency, or patients using tools that improve access to prompt advice and care. Most clinicians do not currently use AI tools in their daily practice, and perspectives on AI interventions vary considerably [124]. While AI is felt to offer significant potential value in improving education and training, automating repetitive tasks, and improving patient outcomes, concerns persist regarding opaque decision-making processes as many models do not offer interpretability of how or why their outputs are generated [125]. In addition, clinical stakeholders highlight inadequate workflow integration and potential to increase rather than alleviate clinical workloads, particularly if models are encouraged to pursue a maximalist approach to investigation and treatment in the name of safety—for fear of criticism that tends to be levelled at omitting active management rather than over-investigating or overtreating [105,126]. Questions around clinical liability, potential impacts on service demand, and long-term implications for workforce planning also remain unresolved [124]. Hype surrounding LLM chatbots has led to discussions about the prospect of clinicians being replaced by AI, although this seems unlikely to happen suddenly with currently available technology [127].

For patients, concerns remain around the risk posed by decision errors and algorithmic biases, reduced transparency in explanations and inadequate rationale for AI-derived recommendations, and potential dehumanisation of the clinician-patient relationship. These challenges are especially relevant in mental health and social care contexts, where interpersonal dynamics are central to care delivery [128]. Nevertheless, patients also recognise potential benefits, acknowledging that AI may improve healthcare accessibility, and increase confidence in medical decisions through access to a ‘second opinion’ [124]. Generative AI applications may lead to progression from existing self-directed research by patients from search engines (the ‘Dr. Google’ phenomenon). While concerns have been raised about the potential of these applications to induce anxiety and reduce trust in clinicians, survey data suggest that access to information promotes confidence in the therapeutic relationship as well as patient-centred care [129,130].

At an organisational and managerial level, healthcare systems face considerable challenges in the selection and implementation of AI models. Limited interoperability between existing technical systems creates integration barriers, while the specificity of current models—often fine-tuned to specific scenarios—restricts broader application. The complexity of managing multi-agent systems, staff training requirements, and inadequate digital infrastructure further complicates successful adoption. Additionally, evolving regulatory frameworks and legislation introduce uncertainties that may slow implementation timelines.

### Governance structures and accountability

VLMs present distinct governance challenges compared to single-purpose AI tools due to their variable and less rigidly defined capabilities—ranging from image interpretation and report generation to clinical decision-support [70]. This versatility makes traditional regulatory approaches, which typically classify medical software by specific functions under a specific ‘intended use’, inadequate for VLMs that can dynamically shift between roles even within a single model or implementation.

Currently, the International Medical Device Regulatory Forum (IMDRF) risk classification, which underlies the FDA and EU MDR standards, uses a simple three-by-three matrix for classification of software risks depending on the significance of the information provided to a healthcare professional, and the clinical risk inherent in the clinical situation or patient (Table 1) [131]. Because VLMs can serve multiple distinct functions, clear delineation of when and how their capabilities are deployed—as well as whether functions are autonomous or semi-autonomous—is required. A comprehensive taxonomy of VLM behaviours is urgently needed to help develop governance frameworks that address their multi-functional nature, ideally fitting into existing classification systems [132].

**Table 1:** Classification of software as a medical device based on the International Medical Device Regulators Forum proposed framework. Classification is based on fixed definitions of the clinical situation the application is intended to be used in, as well as the effect of information generated by the device. VLM applications defy rigid definitions as they may be used in a multitude of scenarios and can have variable effects on clinician action and decision-making.

		Significance of the information provided by the software related to diagnosis, investigation, or treatment		
High: diagnose or treat *~IMDRF 5.1.1*	Medium: influence management *~IMDRF 5.1.2*	Low: inform management *everything else*
**State of clinical situation or patient condition**	Critical *~IMDRF 5.2.1*	Class III *Category IV.i*	Class IIb *Category III.i*	Class IIa *Category II.i*
	Serious *~IMDRF 5.2.2*	Class IIb *Category III.ii*	Class Iia Category II.ii	Class IIa *Category II.ii*
	Nonserious *everything else*	Class IIa *Category II.iii*	Class IIa *Category I.iii*	Class IIa *Category I.i*

In addition, the adaptability of certain VLMs challenges existing regulatory and assurance paradigms. Foundation models such as Google’s Med-PaLM attained near-expert clinician abilities to answer questions about healthcare after being fine-tuned on just 65 question-answer pairs [35]. These models can also improve performance in given tasks after being shown examples of appropriate and successful responses (‘in-context learning’) [133]. This upends machine learning assurance frameworks which often place a significant emphasis on mitigating biases through assurance of training datasets, rather than runtime assurance in a real-world environment [134,135]. The complex interaction between developers designing these versatile foundation models, healthcare institutions or smaller teams deploying them across multiple use cases, and clinicians who may use different functions within the same VLM requires transparent documentation of model boundaries and limitations across its range of capabilities. Lessons may be learned by LLM applications that gain regulatory approval such as Prof Valmed, which took an iterative approach to first establish response consistency through repeated prompting, then clinical efficacy by testing against expert clinicians, and finally real-world piloting to establish safety. Work by POLARIS-GM (Partnership for Oversight, Leadership, and Accountability in Regulating Intelligent Systems: Generative Models in Medicine) is ongoing to define and mitigate the risks of these flexible systems [136]. Regulators should ideally work directly with stakeholders to ensure that innovation is not stifled by reliance on outdated and unaccommodating guidelines—while robustly protecting patients from harm—and that new frameworks have the flexibility to cope with rapidly shifting technological paradigms.

## Conclusion

VLMs provide a technical basis for broadening the capabilities of medical AI to deal with multimodal information, which applies to most areas of clinical practice. Their potential utility has grown since the emergence of transformer architectures that–with sufficient training–can exhibit useful abilities outside tasks they have been exposed to previously. Future applications may range from report generation to triaging of clinicians’ workload and even automated consultation and risk stratification. Validation requirements differ according to the intended use case and degree of autonomy afforded to an AI intervention, but robust study of benefits and risks is essential to ensure that resources are directed efficiently, and that patients and practitioners benefit from change. Ongoing consultation with stakeholders including clinicians, patients, and regulators is necessary to determine how VLMs should be implemented in healthcare, and to develop a permissive environment for innovation.
